# Synergistic surface modification of zirconia nanotubes with silver and hydroxyapatite for enhanced early-stage cytocompatibility: ToF-SIMS insights into fibronectin adsorption

**DOI:** 10.1039/d6ra00504g

**Published:** 2026-07-02

**Authors:** Gabriel Onyenso, Lu Yuan, Nastaran Farahbakhsh, Setareh Orangpour, Paola Carolina Zamudio Franco, Johannes Schmitt, Carsten Engelhard, Christian Pritzel, Henny C. van der Mei, Manuela S. Killian

**Affiliations:** a Chemistry and Structure of Novel Materials, Department of Chemistry & Biology, University of Siegen Paul-Bonatz-Str. 9-11 57076 Siegen Germany Manuela.Killian@uni-siegen.de; b University of Groningen, University Medical Center of Groningen, Department of Biomaterials & Biomedical Technology Antonius Deusinglaan 1 9713 AV Groningen The Netherlands; c Federal Institute for Materials Research and Testing (BAM) Richard-Willstätter Str. 11 D-12489 Berlin Germany; d Analytical Chemistry, Department of Chemistry & Biology, University of Siegen Adolf-Reichwein-Str. 2 57076 Siegen Germany; e Center of Micro- and Nanochemistry and (Bio-)Technology (Cµ), Department of Chemistry-Biology, University of Siegen Germany

## Abstract

Implant surfaces with multifunctional capabilities that can simultaneously facilitate host cell adhesion and proliferation while minimizing bacterial attachment are essential for the long-term success of an implant. In this study, we present a strategy to improve the early-stage cytocompatibility of silver-modified zirconia nanotubes, a key step to optimizing the surface of zirconia nanotubes to achieve potential dual functionality, by co-modification with silver and hydroxyapatite (Hap) nanoparticles. A novel and straightforward strategy to partially coat zirconia nanotubes (ZrNT) with Hap nanoparticles in a manner that still preserves an accessible nanotubular morphology was introduced. The surface morphology and chemical composition were characterized using SEM, XPS, and ToF-SIMS combined with principal component analysis. Protein adsorption studies reveal that coating with Hap enhances fibronectin adsorption, and the detection of RGD-related fragments *via* ToF-SIMS provides an analytical approach for fibronectin identification. Biological assays confirmed that the increased fibronectin adsorption due to Hap coating translates to significantly improved osteoblast adhesion and proliferation. Furthermore, incorporating Hap nanoparticles onto silver-modified ZrNT helped mitigate silver-induced cytotoxicity, thereby improving cytocompatibility. These modifications illustrate a simple yet effective approach toward enhancing the biological performance of these Ag-modified zirconia nanotubes and advance the design towards multifunctional, cytocompatible implant interfaces.

## Abstract 2

Hydroxyapatite modification of Ag-zirconia nanotubes enhances fibronectin adsorption, identified by unique RGD fragments in ToF-SIMS, while preserving the open nanotubular morphology, improves early-stage osteoblast adhesion and cytocompatibility.

## Graphical abstract

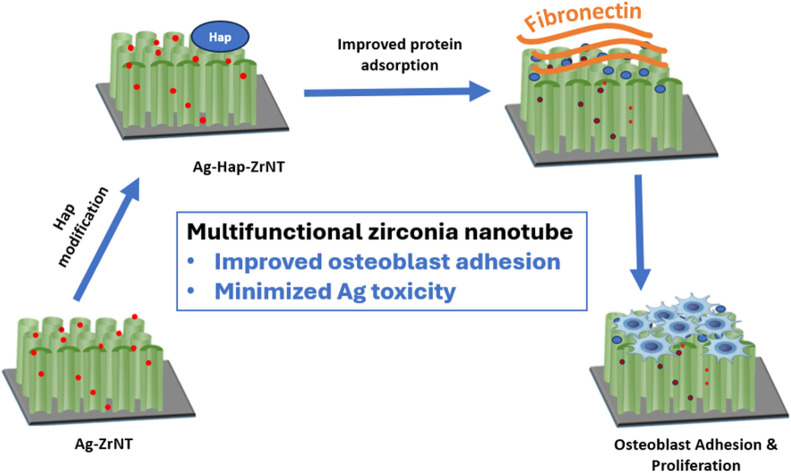

## 1 Introduction

The surface of a biomaterial is the first to make contact with the human tissue, and the nature of this interaction is essential in the success of an implant. In particular, the initial cellular responses such as adhesion, proliferation, and differentiation are critical for tissue integration, ultimately influencing the long-term success of the implant.^1,2^ Therefore, the development of an implant surface that enhances these initial cell responses is essential for achieving an optimal biological outcome.^3–5^ However, bacterial adhesion on the implant surface and subsequent biofilm formation can significantly hinder tissue integration^6^ and once biofilms are formed, the bacteria become highly resistant to the host-defense mechanisms or antibiotics, increasing the risk of biomaterial-associated infection.^6,7^

Currently, most implant materials are designed and evaluated either for their ability to resist bacterial adhesion and biofilm formation^8,9^ or to encourage tissue cell adhesion and proliferation.^10,11^ However, the challenge with such mono-functionalization is that properties that only repel bacteria also tend to negatively affect host cells, and a coating that supports tissue integration may inadvertently allow bacterial attachment.^6,12,13^ This functional overlap underscores the need for implant surfaces with dual functionality, with the ability to minimize bacterial adhesion while simultaneously facilitating host cell adhesion, spreading, and growth.^6,14^

The coating of nanoporous/nanotubular metal oxide-based material on the implant is currently being investigated as a promising candidate to meet these multifunctionality requirements.^15–17^ These nanostructures offer several advantages, such as their chemical stability, good wettability, and biocompatibility.^18–21^ Furthermore, they contain large inner volumes with distinct inner and outer surfaces, which provide the possibility for modification with a range of bioactive molecules and release in a controlled manner to achieve varying and tunable functionality.^22–24^ However, further functionalization is often required to optimize biological performance.

Silver has long been known for its broad-spectrum antibacterial properties and is currently used in burn dressings (as silver salts) and various medical devices/materials to prevent infections.^25,26^ Compared to other forms of silver, silver nanoparticles (AgNPs) have some advantages because they provide a high surface-area-to-volume ratio and a greater proportion of surface atoms, which enhances the antimicrobial activity of silver, even at low concentrations.^27,28^ Additionally, as nanoparticles, they can be effectively anchored within the pores of nanostructured materials, like zirconia nanotubes (ZrNT) to attain a controlled and sustained silver ion release for long-term antimicrobial activity.^29^ AgNP-doped hydroxyapatite (Hap) coatings on zirconium were shown to provide antibacterial properties.^30^ In our previous work, we demonstrated an improved antibacterial property of ZrNT when decorated with AgNPs in an intrinsic one-pot anodization mechanism.^31^ While Ag exhibits exceptional broad-spectrum antibacterial activity, it cannot be regarded as an optimal antibacterial agent due to the concerns regarding its associated cytotoxicity. To address this limitation, this study primarily focuses on improving the cytocompatibility of Ag-modified zirconia nanotubes (Ag-ZrNTs) by additional surface modification with hydroxyapatite nanoparticles (Hap-NPs). The proposed modification strategy is particularly novel in that it still preserves the open morphology of the nanotube, thus maintaining its capacity for potential drug loading for other advanced functionalities. This contrasts with other approaches of modification, where the nanotubes are completely covered after coating with Hap, limiting their utility for controlled drug delivery applications.^29,32–34^

Hydroxyapatite, due to its chemical similarity to the mineral component of natural bone, is widely used in orthopedic procedures as an implant coating to optimize bone ingrowth.^35,36^ Additionally, the calcium–phosphate in Hap has been shown to enhance the expression of the osteoblast phenotype, which can be beneficial for host tissue integration.^36–38^

Using Time of Flight Secondary Ion Mass Spectrometry (ToF-SIMS) combined with Principal Component Analysis (PCA), we further investigated protein adsorption on the zirconia substrates, specifically fibronectin, and demonstrated that the incorporation of Hap increases the exposure of the Arginine-Glycine-Aspartic acid (RGD) amino acid sequence on a surface,^33^ which plays a critical role in cell adhesion.^4,34^ This enhanced RGD motif adsorption may contribute to improved tissue integration.^39,40^

While Ag-containing ZrNT coatings have been shown to provide antibacterial properties, the present study does not directly focus on antibacterial activity of the Hap-coated samples. Instead, the primary focus is on mitigating silver-induced cytotoxicity and enhancing early-stage cytocompatibility of Ag-ZrNT substrates. This work demonstrates that the release of active species from the nanostructure is not obstructed by the chosen coating, providing the foundational basis for future studies aimed at multifunctional implant surfaces.

## 2 Materials and methods

### 2.1 Fabrication of zirconia nanotubes (ZrNTs) and composite nanostructures

Zirconium foil (99.2% purity, 0.1 mm thickness, HMW Hauner, Germany) and zirconium/silver (Zr–Ag) alloy foil (∼1 wt% Ag, 0.1 mm thickness, HMW Hauner) were ultrasonicated in acetone, ethanol, and deionized water for 15 min each and dried with N_2_ to remove any contaminants on the metal surface before electrochemical anodization.

ZrNTs were fabricated by anodizing the Zr foil for 1 h at a constant 90 V, following a gradual ramping up over 60 s. The anodization was carried out in an electrolyte solution containing 2 wt% NH_4_F (Sigma-Aldrich), 2 wt% H_2_O, and 30 wt% formamide (Carl Roth) in glycerol (Carl Roth),^19^ using a high-voltage potentiostat (Jaissle IMP 88 – 200 PC). The electrochemical cell consists of a circular working area of 10 mm^2^ and a platinum counter electrode in a typical two-electrode setup. Post-anodization, the samples were rinsed with deionized water and soaked in ethanol for 2 h to dissolve remains from the organic electrolyte, followed by annealing at a constant 350 °C after a gradual ramp-up over 2 h. The annealing step was performed to remove fluoride ions present in the nanotubes and to improve the phase crystallinity.^41^ The slow ramp-up was necessary to prevent the detachment of the nanotubes from the Zr substrate.

The silver nanoparticle-modified zirconia nanotubes (Ag-ZrNTs) were fabricated by anodizing the Zr–Ag alloy under identical conditions as described above for ZrNT fabrication.^31^

The zirconia compact oxide (Zr–Co, *cf.* Fig. SI S1) was prepared by anodizing the zirconium foil in 1 M H_2_SO_4_ for 30 min at a constant 30 V. After anodization, the anodized ZrO_2_ was rinsed with water and dried with N_2_.

Hydroxyapatite nanoparticles (Hap-NPs) (Sigma-Aldrich) were deposited onto ZrNT and Ag-ZrNT *via* recrystallization. This process involved applying 50 µL of deionized water on the surface of the substrate, followed by the addition of 30 mg of the Hap-NPs. A condition that prevented evaporation of the applied water was created to allow for the solvation and recrystallization of the Hap-NPs on the nanostructured surface. This was achieved by incubating the sample in a water bath at room temperature in a closed system, maintaining a water vapor concentration gradient between the substrate and its surroundings. The substrate was kept at room temperature for 24 h, after which it was washed with deionized water to remove the non-adsorbed Hap and subsequently dried with N_2_.

### 2.2 Protein adsorption

The protein adsorption was performed using 50 µg mL^−1^ of bovine serum albumin (BSA; Sigma-Aldrich) and fibronectin bovine plasma (FBP; Sigma-Aldrich). The protein solutions were prepared in phosphate-buffered saline (PBS) at pH 7.7. The protein solution (400 µL) was pipetted onto the zirconia substrates (1 cm^2^) and incubated at 37 °C for 24 h. After incubation, the samples were rinsed with 1 mL of phosphate-buffered saline (PBS) solution to remove any loosely bound protein, then dried with N_2_ and stored for Tof-SIMS analysis.

### 2.3 Ag^+^ release studies

To investigate the silver release characteristics for HAP-Ag-ZrNT, they were immersed in 10 mL of phosphate-buffered saline (PBS) at 37 °C. After 24 h storage in the dark, the complete solution was removed for analysis, and 10 mL fresh PBS was transferred back into the container with the sample substrates. Release from the samples was monitored for 6 days to mimic the physiological conditions inside the human body. The obtained immersion liquids of each day were stored in the fridge before characterization using inductively coupled plasma mass spectrometry (ICP-MS).

Prior to the measurement, samples were filtered with 0.22 µm nylon syringe filters (1ANX.1, Carl Roth GmbH + Co. KG, Karlsruhe, Germany). All experiments were carried out on a model iCap Qc ICP-Q-MS (Thermo Fisher Scientific, Bremen, Germany). Qtegra ISDS software (2.23.6178.144 (64 bit), Thermo Fisher Scientific) was used to control the instrument. Sample introduction was achieved with a model ESI SC-2 DX autosampler (ESI Elemental Service & Instruments GmbH, Mainz, Germany), a MicroFlow PFA-ST nebulizer (Thermo Fisher Scientific) with a sample flow rate of about 476 µL min^−1^, and a Peltier-cooled cyclonic quartz spray chamber (cooled to 3 °C). The plasma torch injector inner diameter was 1 mm, and the sampling position was set to 3 mm. The high-sensitivity skimmer cone insert (model 2.8, Glass Expansion, Melbourne, Australia) for the nickel skimmer cone was used. The radio frequency (RF) generator power was set to 1400 W, and the extraction lens 1 and 2 voltages were 0 V and −220 V, respectively. Argon (5.0, Messer Industriegase GmbH, Siegen, Germany) was used as cooling gas, nebulizer gas, and auxiliary gas with flow rates of 14 L min^−1^, 0.51 L min^−1^, and 0.8 L min^−1^, respectively.

ICP-MS samples were analyzed with *n* = 10 replicates. To prevent analyte carry-over from sample to sample, the introduction system was rinsed with 2% HNO_3_ in between samples. Every two samples, a measurement of a blank or bi-distilled water was performed to check for carry-over.

^109^Ag^+^ was selected for silver quantification, considering sample matrix interferences (*e.g.*, ^91^Zr^16^O^+^ interference on ^107^Ag^+^) and using mathematical interference correction. Reported values correspond to the overall ICP-MS-available fraction of silver in the samples after filtration.

### 2.4 Material characterization

The surface morphology of the fabricated nanomaterials was examined using an environmental scanning electron microscope (ESEM; Quanta FEG 250 FEI) at low vacuum and 30 kV accelerating voltage. The surface chemical composition was investigated using X-ray photoelectron spectroscopy (XPS/ESCA; SSX-100 S-probe) with monochromatized Al Kα radiation. Core-level binding energies were normalized using the reference C 1s peak set at 284.8 eV. Time of flight secondary ion mass spectrometry (ToF-SIMS) was conducted to obtain high-sensitivity surface chemical information. Positive and negative static SIMS spectra were recorded (ToF-SIMS 4, IONTOF GmbH) using a 25 keV Bi^+^ ion beam bunched down to <0.8 ns and rastered over a 100 × 100 µm^2^ area. For the protein adsorption studies, the peak lists used for spectral evaluation are based on the work of Killian *et al.*^42^ Signals are identified using the accurate mass as well as their isotopic pattern. Principal component analysis (PCA) of the spectra was conducted with the software Spectragui (NESAC/BIO; UW Seattle); the data were normalized and square-root mean-centered. X-ray diffraction measurements (XRD; X'Pert Pro, PANalytical with Cu Kα radiation) were performed on the Hap-modified samples to determine the crystalline phases.

### 2.5 Cell culture and seeding

Human osteoblast-like cell line U-2 OS was purchased from the American Type Culture Collection, and cultured in Dulbecco's Modified Eagle Medium containing 1 g L^−1^ glucose (DMEM-LG; Gibco) supplemented with 10% (v/v) of fetal bovine serum (FBS; Gibco) in a humidified incubator at 37 °C with 5% CO_2_. Cells were detached from the culture flask surface by 0.05% trypsin upon reaching 70% to 90% confluency, and neutralized with fresh culture medium. Cells were collected by centrifugation (1000 rpm, 5 min), and the pellet was resuspended in fresh culture medium. Cell number was determined using a Bürker–Türk counting chamber after staining with trypan blue, and the suspension was diluted in growth medium to a density of 5 × 10^4^ cells per mL prior to seeding onto the samples. Before cell seeding, samples were sterilized with ultraviolet irradiation (UV-C, wavelength of 254 nm, 8 W) for 30 min on each side. Subsequently, 1.5 mL of cell suspension was added to each well (12-well plate) containing samples, yielding a seeding density of 2 × 10^4^ cells per cm^2^.

### 2.6 Cytocompatibility studies

After culturing for 1 and 3 days, cell viability and cell morphology were evaluated with an XTT assay and the confocal laser scanning microscope (CLSM), respectively. For the XTT assay, cells were incubated with 1 mL fresh culture media and 0.5 mL of activated XTT reagent (Thermo Fisher) at 37 °C, 5% CO_2_ in an incubator for 2 h. After incubation, the media were gently mixed and transferred to a 96-well plate. The absorbance was measured with dual wavelengths at 450 nm (formazan product) and 660 nm (background correction) using a microplate reader (Biotek Synergy H1), and the culture medium containing activated XTT but no cells was used as a blank control. The absorbance of the samples was calculated using the following equation:

1 Abs (sample) = [Abs_450 nm_ (sample) − Abs_450 nm_ (blank)] − Abs_660 nm_ (sample)

The cell viability was expressed as a percentage relative to the ZrNT control on day 1, which was set to 100%:2
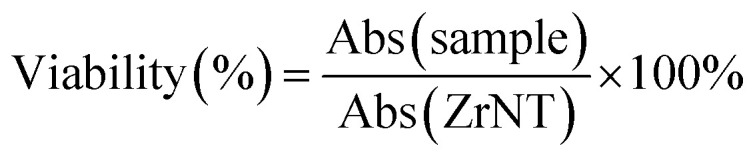


To visualize cell morphology on the samples, cells were stained with Phalloidin-FITC (Abcam) for staining the cytoskeleton and DAPI (Thermo Fisher) for staining the nucleus and imaged with CLSM (Leica Stellaris 5). Briefly, cells were washed with PBS twice, fixed with 4% formaldehyde for 30 min, and permeabilized with 1% Triton X-100 in PBS for 5 min. Following permeabilization, cells were incubated with 1% bovine serum albumin in PBS (PBSA), then stained with Phalloidin (1 : 1000 in PBSA) and DAPI (1 : 100 in PBSA) for 30 min at room temperature in the dark. Finally, samples were washed twice with PBS and imaged by CLSM (Phalloidin-FITC: excitation/emission 488 nm/490–548 nm; DAPI: excitation/emission: 405 nm/420–470 nm). For each sample, three different locations were imaged, with CLSM region of interest 291 × 291 µm^2^. Cell density and cell surface coverage were quantified from the acquired images using Fiji software.

### 2.7 Statistics

Results were expressed as means ± standard deviation (SD), and analyzed with one-way or two-way analysis of variance (ANOVA), followed by Tukey post hoc test to compare multiple groups using GraphPad Prism 10.5.0 (Dotmatics). Differences between groups at *p* < 0.05 were considered statistically significant.

## 3 Results and discussion

### 3.1 Fabrication of zirconia nanotubes and composite nanostructures

**Fig. 1:**
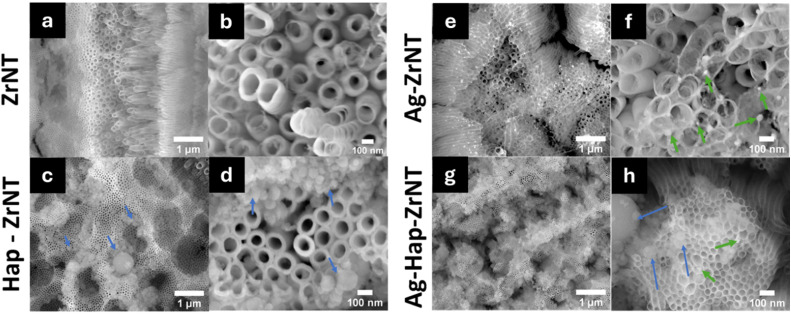
SEM top view image (a) ZrNT (fabricated by anodizing Zr metal), (b) high magnification view of ZrNT, (c) Hap-ZrNT composite nanostructures, (d) high magnification view of Hap-ZrNT. The blue arrows indicate the locations of the hydroxyapatite nanoparticles. (e) Ag-ZrNT fabricated by anodizing Zr–Ag alloy, (f) high magnification view of Ag-ZrNT, (g) Hap-Ag-ZrNT composite nanostructures, (h) high magnification, the green arrows indicate the locations of silver and the blue arrows the location of hydroxyapatite nanoparticles.

Zirconia nanotubes (ZrNT) were obtained by electrochemical anodization of zirconium foil in a fluoride-containing electrolyte at 90 V for 1 h. The SEM top-view images of the fabricated ZrNT and the hydroxyapatite-modified nanotubes (Hap-ZrNT) are shown in Fig. 1a–d. They reveal a highly ordered and densely packed nanotubular structure with an average diameter of 100 ± 10 nm, with some regions also displaying smaller diameters. Similarly, the SEM image of Hap-ZrNT displays well-ordered, packed, and open nanotubes with comparable diameters. In addition to the nanotubes, hydroxyapatite nanoparticles are visible along the surface, primarily as aggregates, but do not entirely cover the underlying nanostructure.

Zirconia nanotubes intrinsically modified with silver nanoparticles (Ag-ZrNT) were synthesized by electrochemical anodization of zirconium–silver alloy.^31^ Subsequently, they were co-modified with hydroxyapatite nanoparticles to obtain Hap-Ag-ZrNT. The SEM images in Fig. 1e–h show the top view of both samples. At higher magnification, a highly ordered and densely packed nanotubular structure is clearly visible, with slightly larger variations in diameter. The arrows indicate the location of the Ag and Hap NPs.

**Fig. 2:**
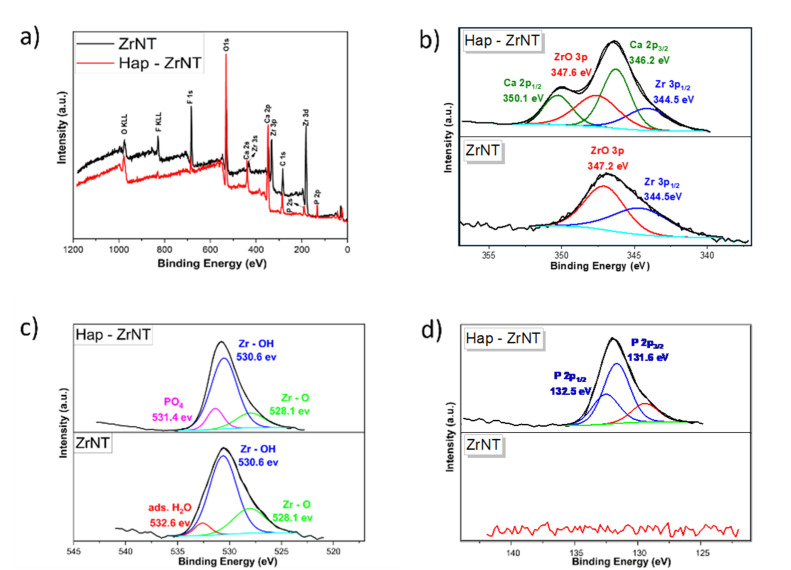
(a) XPS survey of ZrNT and Hap-ZrNT, high-resolution XPS spectra of ZrNT and Hap-ZrNT (b) Ca 2p, (c) O 1s, (d) P 2p.

X-ray photoelectron spectroscopy (XPS) analysis was performed to evaluate the coating deposited under Hap exposure, *cf.*Fig. 2. A typical survey XPS spectrum from the ZrNT samples is shown in Fig. 2a. The expected Zr 3d and O 1s peaks are present in the ZrNT sample, in addition to a F 1s peak originating from ammonium fluoride in the anodization electrolyte. Despite post-treatment annealing steps intended to reduce electrolyte-related contamination, residual fluoride impurities remained detectable. The F-ion content can be significantly lowered compared to non-annealed samples,^18^ however, the gentle annealing conditions chosen in this work may not be sufficient to completely remove the electrolyte residues from the nanostructure, as previously observed for other oxides.^41^ For the Hap-ZrNT sample, in addition to the Zr-related peaks, characteristic peaks of hydroxyapatite, such as Ca 2s, Ca 2p, P 2s, and P 2p, are also observed. To obtain more details about the chemical composition of the samples, high-resolution analysis of the selected peaks was performed. Fig. 2b presents the high-resolution Ca 2p spectra from ZrNTs with and without Hap deposition. In the Hap-ZrNT sample, the Ca 2p signal shows a doublet peak located at 350.1 eV (2p_3/2_) and 346.2 eV (Ca 2p_1/2_), with a binding energy (BE) separation of 3.9 eV. Both the peak position and BE differences are in good agreement with the values reported in the literature.^43,44^ Further peaks at 347.2–347.6 eV, assigned to ZrO 3p, and at 344.5 eV, assigned to Zr 3p_1/2_, are also observed in the ZrNT sample.^45^ The Ca 2p doublet is not present in the ZrNT sample. The O 1s (Fig. 2c) spectrum from the ZrNT sample shows a main O 1s peak at 530.6 eV, which typically corresponds to hydroxide bonded to Zr in the ZrO_2_ lattice, along with two additional peaks at 532.6 eV and 528.1 eV, which correspond to possible adsorbed water and Zr–O bonds, respectively.^46,47^ Similarly, multiple peaks were also observed on the Hap-ZrNT sample, with 531.4 eV being assigned to phosphate, providing no distinct spectral differences between the two materials in this region. The P 2p spectrum (Fig. 2d) of the Hap-ZrNT sample confirms the presence of phosphate, showing the typical doublet peaks at 132.5 eV (P 2p_3/2_) and 131.6 eV(P 2p_1/2_). The unassigned signal most likely corresponds to organophosphorous compounds P-C,^18^ potentially originating from contamination. The phosphate-related peaks are absent in the ZrNT sample.

The relative atomic concentration and the calculated Ca/P, O/Ca ratios from the XPS data are presented in Table 1.

**Table 1:** Atomic concentration and ratios from XPS measurement of ZrNT and Hap-ZrNT

Atomic concentration (%)							
	C 1s	O 1s	Zr 3d	Ca 2p	P 2p	Ca/P	O/Ca
ZrNT	36.2	42.3	21.4	—	—	—	—
Hap-ZrNT	20.9	49.4	1.2	13.7	9.1	1.5	3.4

For the ZrNT sample, the atomic concentration (at%) of C, O, and Zr were 36.2, 42.3, and 21.4 at%, respectively, and as expected, there was no detectable amount of P 2p and Ca 2p. In contrast, the Hap-ZrNT showed a significant reduction in the Zr atomic concentration from 21.4 at% in the ZrNT to 1.2 at%, while the Ca 2p and P 2p, not observed in the ZrNT, were 13.7 at% and 9.1 at%, respectively. This is expected due to the hydroxyapatite coating on the HaP-ZrNT, which shadows the underlying zirconium oxide substrate. Considering that the probing depth of XPS is approximately 3–10 nm, the exposure of ZrO_2_ beneath the hydroxyapatite is largely limited. The Ca/P and O/Ca ratios are commonly used to identify the different calcium phosphate phases,^46,48^ and the theoretical values for these ratios are 1.67 and 2.6 for hydroxyapatite; however, 1.5 and 3.4 were obtained. These values agree with other studies that have reported that the XPS-derived Ca/P ratios of the different calcium phosphate phases are consistently lower than the bulk stoichiometric values, while the O/Ca ratios are typically higher.^46^ Chusuel *et al.* suggested that this discrepancy might be due to prolonged exposure of the Hap particles to the X-ray source, which results in decomposition of the Ca substituent in the powder.^49^

ToF-SIMS measurement, combined with principal component analysis (PCA), was performed to further chemically characterize Hap-coatings. Both positive and negative ion spectra were acquired. Selected positive peaks (Ca^+^, CaO^+^, CaOH^+^, ZrO^+^, Ca_2_PO_4_^+^, CaH^+^) and negative peaks (O^−^, OH^−^, P^−^, O_2_^−^, PO^−^, PO_2_^−^, and PO_3_^−^) were normalized to the sum of intensities of all selected peaks to enable a quantitative comparison between the spectra.

**Fig. 3:**
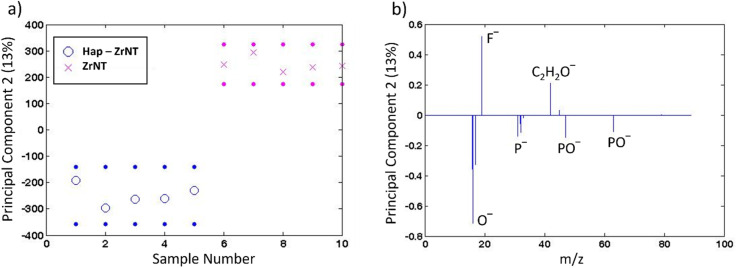
(a) scores plot, (b) loading plot, from PC 2 of the negative ion spectra of hydroxyapatite modified zirconia nanotube (Hap-ZrNT) and bare zirconia nanotube (ZrNT). The dotted lines in the score plot show the 95% confidence limits.

PCA of the negative spectra was able to effectively distinguish between the two surfaces, while positive spectra yielded less clear separations, indicating that the substances responsible for the sample differences preferentially form negative ions. Fig. 3 shows the scores and loadings plots of the negative spectra. The scores plot shows a clear separation of the sample surfaces, while the loadings plot identifies the fragments responsible for the separation. Peaks related to hydroxyapatite (PO^−^, PO_2,_ P^−^) contribute highly to differences and are found mainly on the Hap-ZrNT sample. In contrast, F^−^ and C_2_H_2_O^−^, which are attributed to the electrolyte used in anodization, feature more on the ZrNT sample.

The characteristic fragment signals for ZrNTs along with signals of the hydroxyapatite in the negative and positive spectra, such as *m*/*z* 39.97 (Ca^+^), *m*/*z* 55.94 (CaO^+^), *m*/*z* 56.97 (CaOH^+^), *m*/*z* 46.96 (PO^−^), *m*/*z* 62.97 (PO_2_^−^), *m*/*z* 78.89 (PO_3_^−^), were plotted for both samples (Fig. SI S2 and S3). The latter were detected in the Hap-ZrNT sample but not in the ZrNT sample, confirming the successful coating of the hydroxyapatite on the zirconia nanotubes.

The PO_3_^−^/PO_2_^−^ peak ratio, a previously reported indicator for differentiating calcium phosphate phases, was calculated as 0.20 ± 0.02 for the Hap-ZrNT sample. This value aligns well with previously reported values for hydroxyapatites and further supports the successful modification.^46,49^ Moreover, the ToF-SIMS results are consistent with the findings from XPS analysis.

It has been well reported that surface hydroxyl groups (–OH) of metal oxides (*e.g.*, TiO_2_ and ZrO_2_) can induce apatite formation.^50^ The hydroxyl groups act as nucleation sites where calcium (Ca^2+^) and phosphate ions (PO_4_^3−^) can attach and form apatite. This is due to non-covalent interactions, such as electrostatic interactions (*e.g.*, between Ca^2+^ and surface O^2−^/OH^−^), hydrogen bonding, and van der Waals forces.^51^

Hydration of the Hap particles is an important process for their adsorption onto oxide surfaces. Improved hydration enhances surface chemistry and subsequent reactivity of the calcium Ca^2+^ and phosphate PO_4_^3−^ ions. In this work, the deposition on the metal oxide was achieved by maintaining an aqueous environment that allows Hap nanoparticles to adsorb water molecules onto their surface *via* hydrogen bonding or ion–dipole interactions. This adsorbed water forms a hydration shell around the Ca^2+^ sites and phosphate groups, and this enhances the adsorption and ionic mobility on the ZrNT surface.

Various methods have been used to deposit Hap on metal oxide nanotubes, such as electrochemical deposition, plasma spray, and radio frequency sputtering, generally aimed at improving osteoconductivity and promoting bone integration.^52,53^ These are often combined with pretreatment steps (*e.g.*, annealing, treating with alkaline solution, *etc.*) to improve the interfacial bonding between the nanotubes and the Hap.^54^ However, in many of these reported systems, the deposited Hap layers form dense, continuous coatings that often result in complete coverage of the nanotube openings.^55–57^ Such complete surface coverage can significantly reduce the accessibility of the nanotube's inner volume and limit its utility as a multifunctional platform for secondary loading applications, such as for sustained drug delivery, growth factor incorporation, or combined therapeutic strategies.^22,58,59^

In contrast, the approach introduced in this work intentionally aimed to achieve partial Hap modification while preserving the open nanotubular morphology of the ZrNTs. SEM analysis (Fig. 1 c and d) clearly demonstrates that the Hap nanoparticles are distributed along the nanotube surface primarily as localized aggregates rather than as a dense continuous coating. Importantly, the nanotube openings remain visible after modification, indicating that the nanostructured architecture is retained. This distinction is significant because preserving the open morphology maintains the potential utility of the nanotubes as multifunctional platforms for future therapeutic loading and controlled-release applications.^22^

The preservation of the open nanotubular morphology is also strongly influenced by the amount of Hap nanoparticles used during deposition. In this work, a relatively low amount of Hap was intentionally used to achieve partial surface coverage while maintaining accessibility to the nanotube openings. Experimentally, it was observed that increasing the Hap amount led to significantly denser deposition and complete coverage of the nanotube surface. This behavior is likely associated with increased particle aggregation and enhanced secondary nucleation during recrystallization, leading to the formation of a continuous calcium phosphate layer across the nanotube openings. At lower Hap concentrations, deposition preferentially occurs at energetically favorable surface regions, such as nanotube rims and outer walls, forming localized nanoparticle aggregates while preserving the underlying nanotubular architecture. In contrast, higher particle concentrations promote extensive crystal growth and interparticle bridging, ultimately covering the nanotube morphology. Similar concentration-dependent coating behavior has previously been reported for calcium phosphate deposition on anodic oxide nanostructures, where prolonged deposition times or higher precursor concentrations led to complete pore covering and the formation of dense apatite layers.^32,54,60^

### 3.2 Protein adsorption on zirconia nanotubes

**Fig. 4:**
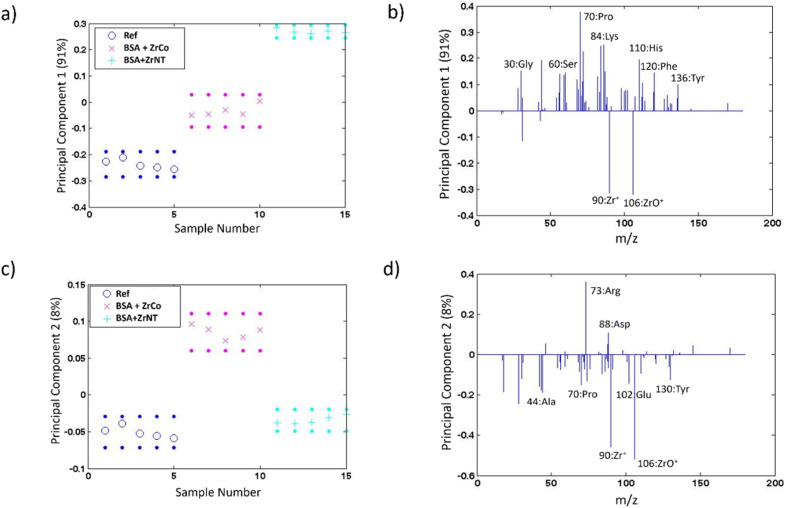
Principal component analysis of ToF-SIMS spectra: (a) and (c) scores plot from PC 1 and 2 of the positive ion spectra of bovine serum albumin (BSA) adsorbed on zirconia nanotubes (ZrNT) and zirconia compact oxide (ZrCo) from 1 mg mL^−1^ protein solution. (b) and (d) corresponding loading plots. The dotted lines in the score plot show the 95% confidence limits. Ref stands for the reference sample (bare ZrNT substrate without protein).

When a surface is in contact with biological media such as body fluids, protein adsorption occurs rapidly and determines, *e.g.*, the body's response to an implant material. Albumin is the most prominent protein in most human body fluids. ToF-SIMS in combination with PCA was used to study the effect of zirconia surface morphology on protein adsorption by analyzing amino acid mass fragments from albumin adsorbed onto ZrNT and compact zirconium oxide (ZrCo) substrates. The PCA scores and loadings from the first two principal components obtained from the ToF-SIMS positive spectra are shown in Fig. 4, and it successfully distinguishes between albumin adsorbed onto ZrNT and ZrCo substrates, suggesting albumin interacts differently with the zirconia substrate depending on its morphology. The scores plot describes the relationship among the samples (spectra) and represents a projection of the original data onto the given principal component axis. The loadings show which variables (amino acid peaks) are responsible for the separation observed in the scores plot. Together, the scores and loadings represent a concise summary of the original data.^42^ The scores plot for PC1 accounts for 91% of the total variance in the spectra. The corresponding loading plots reveal amino acid fragment ions featured predominantly on the modified substrate compared to the reference sample (zirconia substrate without albumin), which mainly displays the characteristic fragment ions of the substrate (*m*/*z* 89.89: Zr^+^, *m*/*z* 105.89: ZrO^+^). PC2, which accounts for the remaining 8% of the variance, differentiates between amino acid fragments featured significantly on the ZrNT compared to the ZrCo. A similar trend was observed at lower concentrations of albumin (50 µg mL^−1^), where PC1 and PC2 captured 86% and 11% of the variance, respectively (Fig. SI S4) indicating that changing the concentration of albumin in solution affects how it attaches to the surface.

**Fig. 5:**
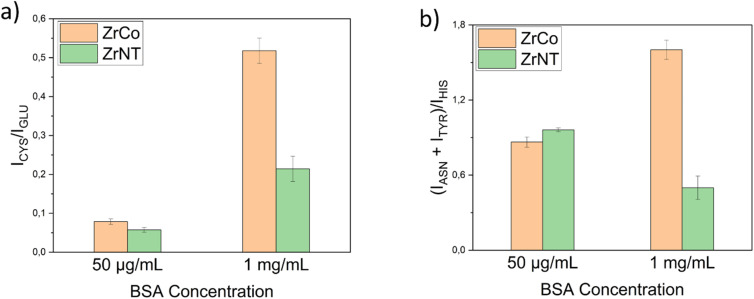
Tof-SIMS peak intensity ratios of selected amino acids from bovine serum albumin (BSA) adsorbed on zirconia nanotubes (ZrNT) and zirconia compact oxide (ZrCo) at different concentration. The intensity ratio used as an indicator of protein denaturation (a) and orientation (b), respectively, suggest the extent of denaturation and orientational changes of BSA adsorbed on ZrNT and ZrCo. The ratios were calculated by normalizing the intensities of the selected amino acids to the total intensity of the spectrum to correct for differences in total secondary ion yield from spectrum to spectrum. Error bars represent standard deviations, *n* = 5.

To probe the nature of albumin adsorption on these substrates, it is important to ascertain the level of protein denaturation. The spatial conformation of a protein is critical to its biological function, and loss of this structure can significantly affect its activity.^42,61^ Trends in specific peak ratios of amino acids that are unevenly distributed in proteins have been previously analyzed to provide structural information about proteins immobilized on surfaces.^62^ To estimate the extent of denaturation, the peak intensity ratio of cysteine to glutamic acid on the substrate was evaluated (Fig. 5a). These amino acids were chosen based on the crystal structure of BSA, in which cysteine residues are generally within the protein core, while glutamic acids are typically surface exposed. Thus, the ratio can be examined as an indication to estimate the level of denaturation. An increase in this ratio indicates an increase in albumin denaturation.^61^ The ratio was higher on ZrCo than on ZrNT for the highest concentration of BSA, indicating a higher degree of albumin denaturation on compact oxide. At lower BSA concentrations, the differences in ratio were smaller; however, the overall ratio was higher on the substrates from higher concentrated solutions. This suggests that albumin denatures more on compact oxide and that denaturation increases with packing density at higher surface concentrations. Previously, a beneficial effect of oxide nanotubes on protein activity has been demonstrated, presumably due to the retention of water within the nanotubes and, consequently, a prevention of drying of surface immobilized proteins.^42,63^ To investigate the influence of surface morphology on the protein orientation, a similar analysis of the amino acid ratio was done. This aimed to determine whether the protein adopts different orientations depending on the surface morphology. The amino acids were selected based on their known positions in the 3D structure of BSA, and the ratio was calculated according to the equation (*I*_Asn_ + *I*_Tyr_/*I*_His_).^61^ The resulting amino acid ratios were different for each surface (ZrCo *vs.* ZrNT), indicating that albumin orients differently depending on the surface morphology (Fig. 5b). The ratios also seem to change with protein concentration, suggesting that the concentration influences how the protein orients on the surface.^64^

The nature and activity of protein adsorption on a surface play a critical role in mediating the initial cellular response to an implant material.^1^ Albumin is the most abundant serum protein, and its adsorption properties have been widely studied on various surfaces.^65,66^ Improved albumin adsorption on the nanotubes compared to the compact oxide can be attributed to the increased surface area of the nanotubes. ZrNT offer more active sites for protein adsorption, including both the inner and outer walls, which are unavailable on flat compact oxide. Additionally, better protein adsorption may also result from the physical entrapment of the proteins within the zirconia nanotubes.^63,67^ These effects become more pronounced at higher concentrations, where steric repulsion between protein molecules plays a more significant role, leading to greater differences in both denaturation and changes in the orientation of the protein across the two substrates. These results are consistent with previous findings on protein adsorption on other nanostructured metal oxides, where surface area, in addition to other factors like surface charge density and size, was shown to influence protein–surface interaction.^42,68^

### 3.3 Fibronectin adsorption and identification using ToF-SIMS and PCA

ToF-SIMS combined with PCA was additionally used to characterize fibronectin on zirconia substrates (Fig. SI S5). Fibronectin was selected due to its key role as an adhesive protein in the extracellular matrix, facilitating cell attachment and spreading.^69^ While fibronectin adsorption on surfaces has been previously studied with ToF-SIMS, there have been limited investigations to compare the fragment pattern of multiple proteins to identify specific fragment markers for fibronectin. Wagner and Castner performed PCA on ToF-SIMS spectra of various proteins to distinguish the amino acid fragment pattern, while Tidwell *et al.* compared the distinct amino acid fragmentation patterns for albumin and fibronectin and were able to identify that amino acids like alanine, lysine, and leucine/isoleucine are more abundant in albumin, while proline is more abundant in fibronectin.^70,71^ Although these findings provide valuable insight into detecting differences in protein adsorption across complex samples and mixtures, there is also a need for other definite signals for identifying proteins, especially from multi-component protein solutions.

**Fig. 6:**
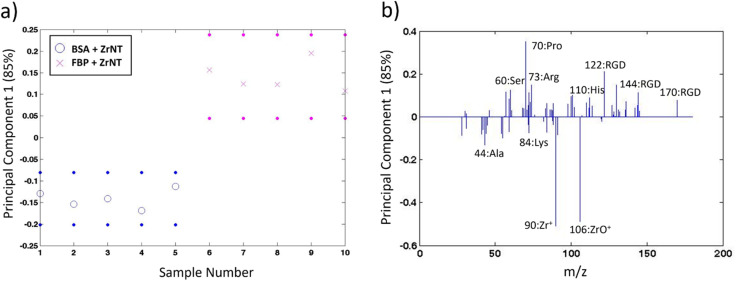
(a) Scores plot of the positive ion spectra of bovine serum albumin (BSA) and fibronectin bovine plasma (FBP) adsorbed on zirconia nanotubes (ZrNT) from 50 mg mL^−1^ protein solution; (b) corresponding loading plots. The dotted lines in the score plot show the 95% confidence limits.

**Fig. 7:**
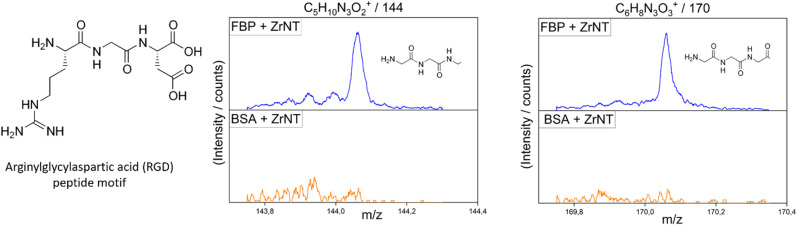
Comparison of Tof-SIMS of specific arginyl-glycyl-aspartic acid (RGD) peptide motif fragment ions on fibronectin bovine plasma (FBP) adsorbed on zirconia nanotubes (FBP + ZrNT), and bovine serum albumin (BSA) adsorbed on zirconia nanotubes (BSA + ZrNT).

Fig. 6 shows the scores and loadings of PCA of Tof-SIMS spectra from fibronectin and albumin adsorbed on ZrNT. PC1 distinguishes the samples based on the adsorbed protein. The loading plots indicate that the amino acid fragments C_2_H_6_N^+^ (44.05, Ala), C_3_H_4_N^+^ (54.03, His), C_5_H_10_N^+^ (84.08, Lys), and C_4_H_10_N^+^ (72.08, Val) feature predominantly on the albumin-coated substrate. In contrast, several amino acid peaks such as C_4_H_8_N^+^ (70.07, Pro), C_3_H_8_NO^+^ (74.06, Thr), C_3_H_4_O_3_^+^ (88.02, Asn), in addition to non-amino acid peaks (C_3_H_12_N_3_O_2_^+^/122.09, C_5_H_10_N_3_O_2_^+^/144.08, C_6_H_8_N_3_O_3_^+^/170.06) contribute most significantly to the fibronectin spectra. The non-amino acid peaks are not related to standard amino acid fragments; rather, they correspond to the tripeptide arginyl-glycyl-aspartic acid (RGD), a well-known motif involved in cell adhesion to the extracellular matrix (ECM). This sequence is found in adhesive proteins such as fibronectin, vitronectin, and fibrinogen, but not in albumin. Fig. 7 displays the ToF-SIMS spectra of RGD fragment signals on albumin and fibronectin modified substrates. RGD signals are clearly present on the fibronectin-modified surface but are absent on the albumin-modified ZrNTs.

Therefore, these RGD-related fragments may serve as ToF-SIMS unique molecular markers for fibronectin when compared to albumin (or any other protein without this motif). Moreover, since the RGD motif is recognized by cell receptors that mediate adhesion, its exposure to biomaterial surfaces may serve as an indicator of biocompatibility. A surface that promotes the presentation of the RGD motif in the correct orientation may be considered more favorable for supporting cellular interactions and tissue integration.

### 3.4 Effect of hydroxyapatite coating on fibronectin adsorption

**Fig. 8:**
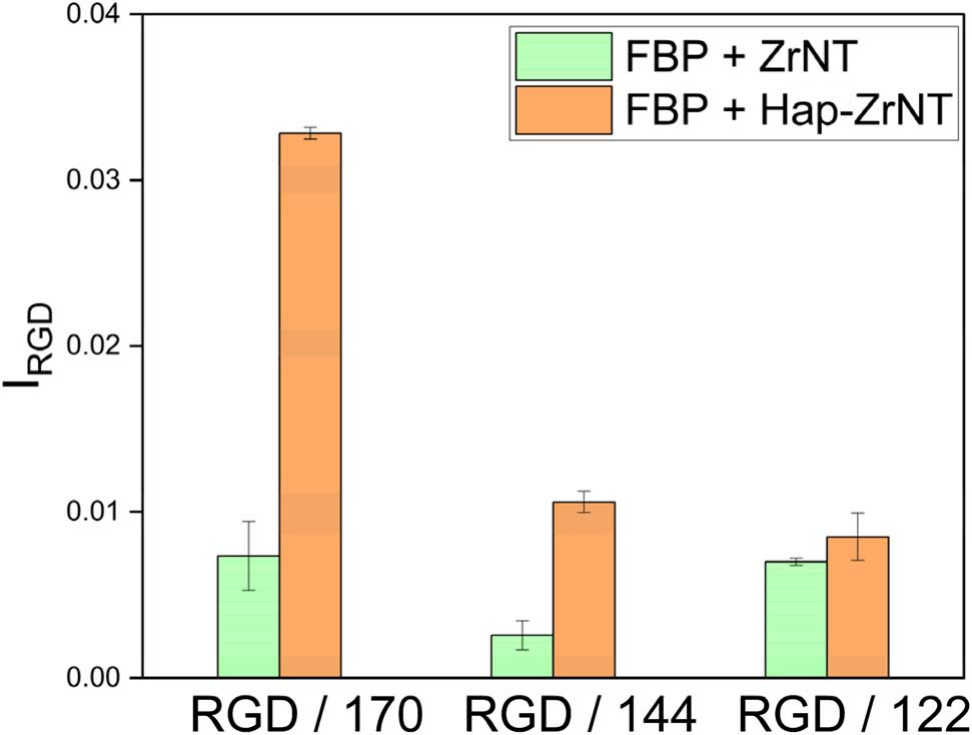
ToF-SIMS normalized peak intensity of the RGD-related fragments (C_5_H_10_N_3_O_2_^+^/144.08, C_6_H_8_N_3_O_3_^+^/170.06, C_3_H_12_N_3_O_2_^+^/122.09) on the Hap-modified ZrNT and unmodified ZrNT. An increased adsorption on the Hap-modified ZrNT is indicated. Error bars represent standard deviations, *n* = 3.

To investigate the influence of a Hap coating on fibronectin adsorption, ToF-SIMS analysis of the RGD fragments was carried out on both Hap-ZrNT and bare ZrNT. Fig. 8 compares the normalized peak intensities of RGD signals on both surfaces and indicates an increase in fibronectin adsorption on the Hap-modified surface. All the RGD-related fragments (C_13_H_12_N_3_O_2_^+^/122.09, C_5_H_10_N_3_O_2_^+^/144.08, C_6_H_8_N_3_O_3_^+^/170.06) exhibit increased peak intensity on the Hap-ZrNT sample.

Hydroxyapatite is a biocompatible ceramic, and its biocompatibility is attributed to its ability to enhance protein adsorption.^72,73^ El-Ghannam *et al.* demonstrated that a calcium–phosphate layer coated on bioactive glass selectively attracts high molecular weight serum proteins such as fibronectin, which subsequently facilitates cells, such as osteogenic (bone forming) cells, to attach and spread on the surface.^74^

Calcium phosphate surfaces are typically negatively charged under physiological conditions,^75^ while fibronectin is a large glycoprotein with regions (domains) enriched with clusters of positively charged amino acids.^76^ Electrostatic interaction can therefore occur between the oppositely charged groups of the fibronectin molecules and the Ca_*x*_PO_*y*_ surfaces. In addition, Ca_*x*_PO_*y*_ is chemically similar to bone minerals, which naturally bind extracellular matrix proteins like fibronectin. This biomimetic quality can help the fibronectin “recognize” the surface, promoting specific, conformation-preserving interactions.

### 3.5 Effect of hydroxyapatite coating on cell adhesion and proliferation

Previously, we demonstrated a simple strategy for improving the antibacterial properties of ZrNT by functionalization with silver nanoparticles in a one-pot anodization strategy.^31^ Although the concentration of released silver ions was minimal, the antibacterial properties of the Ag-ZrNT were significantly enhanced. However, concerns remain about the potential cytotoxicity of silver ions. Therefore, cell adhesion and proliferation were investigated for ZrNTs and Ag-ZrNTs, as well as their Hap-NP modified counterparts. As demonstrated in the previous section, hydroxyapatite increases the adsorption of cell adhesion proteins, such as fibronectin. Consequently, coating Hap on ZrNT and Ag-ZrNT is expected to improve cell adhesion and proliferation. To confirm this hypothesis, we evaluated the effect of the Hap coating on cell adhesion and proliferation.

**Fig. 9:**
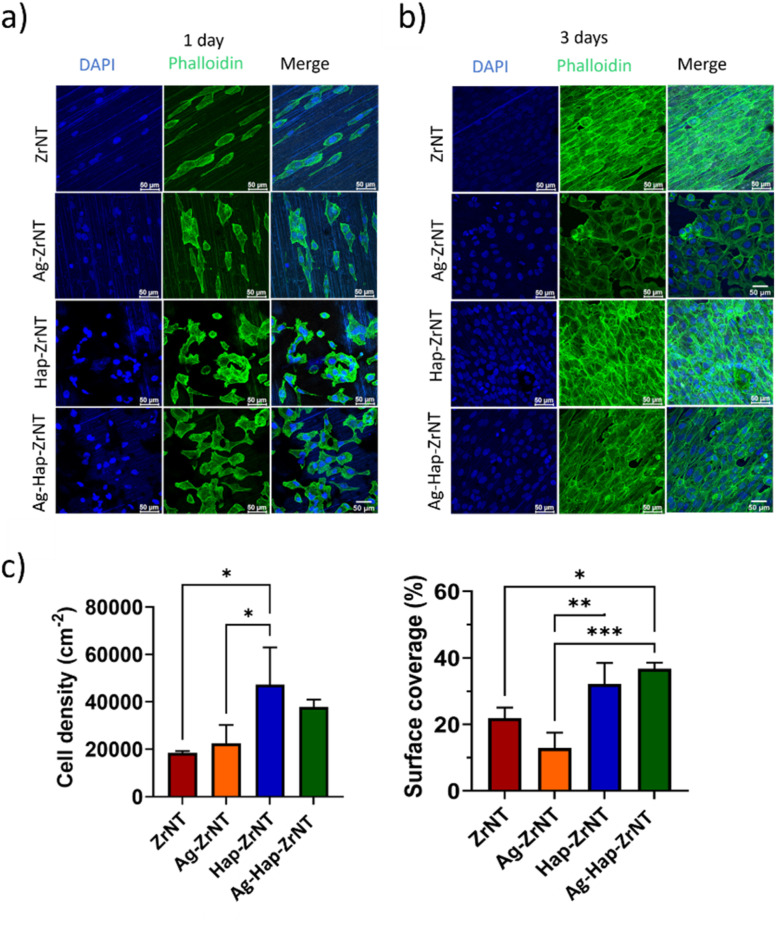
CLSM images of osteoblast cells on ZrNT, Hap-ZrNT, Ag-ZrNT, and Ag-Hap-ZrNT surfaces after cell seeding for (a) 1 day and (b) 3 days. Cytoskeleton stained by Phalloidin (green) and nuclei stained by DAPI (blue). (c) Cell density and surface coverage after culturing on sample surfaces for 1 day. Three representative CLSM images at different locations of samples were used to obtain cell density and surface coverage, analyzed by Fiji software. One-way ANOVA followed by Tukey post hoc test used for multiple groups comparison; *p < 0.05, **p < 0.01, ***p < 0.001.

The CLSM images of the osteoblast cells seeded on ZrNT, Hap-ZrNT, Ag-ZrNT, and Hap-Ag-ZrNT are shown in Fig. 9. The non-anodized foils are shown in Fig. SI S6 in order to demonstrate the influence of the nanostructures on cell fate. After culturing on different surfaces for 1 day, cells on ZrNT and Ag-ZrNT exhibit similar morphology, with actin filaments that are aligned along a specific axis (thin, spike-like structures) and show limited spreading area. In contrast, cells on the Hap-modified substrate display more spreading structures with actin filaments extending in multiple directions, indicating enhanced adhesion. The unidirectional, spike-like actin filament observed on the non-modified ZrNT suggests that the cells are still probing for suitable adhesion sites. On the other hand, the well-spread and branched actin filaments observed on the Hap-modified surfaces suggest a better and more stable cell–substrate interaction. Quantitative analysis confirmed a higher number of adhered cells and a larger spreading area (Fig. 9a and b) on the Hap-modified substrate (Hap-ZrNT and Hap-Ag-ZrNT) compared to the non-Hap-modified ones (ZrNT and Ag-ZrNT), *i.e.*, Hap-coated ZrNTs act more similarly to the non-anodized metal foils (*cf.* Fig. SI S7). Furthermore, cells proliferated after 3 days and fully covered the surfaces except Ag-ZrNT. These findings underscore the positive effect of hydroxyapatite coating on cell adhesion, spreading, and proliferation.

**Fig. 10:**
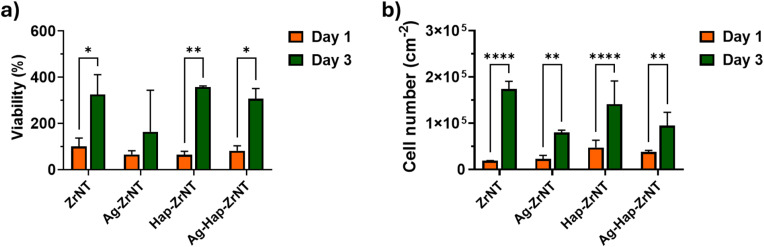
(a) Cell viability of osteoblasts cultured on different ZrNT substrates for 1 and 3 days. Data represent the mean ± s.d. (*n* = 3); values were normalized to the ZrNT group on day 1, which was set to 100%. Two-way ANOVA followed by Tukey post doc test used for multiple groups comparison, which showed the effects of surface (0.0638), time (0.1391), and their interaction (*p* = 0.2058). (b) Cell density on different ZrNT substrates, analyzed by fluorescent images obtained from confocal laser scanning microscopy with Fiji software. Two-way ANOVA followed by Tukey post hoc revealed significant effects of surface (*p* = 0.0007), time (*p* = 0.0090), and their interaction (*p* = 0.0063), indicating that the effect of surface depends on the time point. * denotes significant differences, *p < 0.05, **p < 0.01, ****p < 0.0001.

An XTT assay was further employed to evaluate the cytotoxicity and proliferation of the various zirconia samples. Fig. 10 presents the cell viability results for the different substrates on day 1 and 3, noting that the relatively small sample size (*n* = 3) may limit statistical power. ZrNT and its Hap-modified equivalent exhibited no cytotoxicity. However, Ag-ZrNT showed some cytotoxic effects, probably due to released silver ions, but when co-modified with Hap, the cytotoxic effect was mitigated. All substrates showed substantial proliferation, except for Ag-ZrNT, where no significant proliferation was observed, which was consistent with the CLSM results.

The key takeaway is that Hap-Ag-ZrNT significantly mitigates the cytotoxicity of silver ions, supported by enhanced cell proliferation, even though the mechanism underlying this effect cannot be conclusively determined based on the current data. Possible contributing factors include enhanced adsorption of adhesion proteins (Fig. 8) and modulations of the local cellular microenvironment that favor cell attachment in Hap-rich regions, which is consistent with previous studies showing that Hap actively influences osteoblast attachment and growth.^36^ Additionally, it is well established that silver nanoparticles can induce cytotoxicity through the formation of intracellular reactive oxygen species (ROS).^77^ The mechanism, including ROS-related pathways by which Hap modification influences silver-associated cytotoxicity, will be the focus of future investigation.

**Fig. 11:**
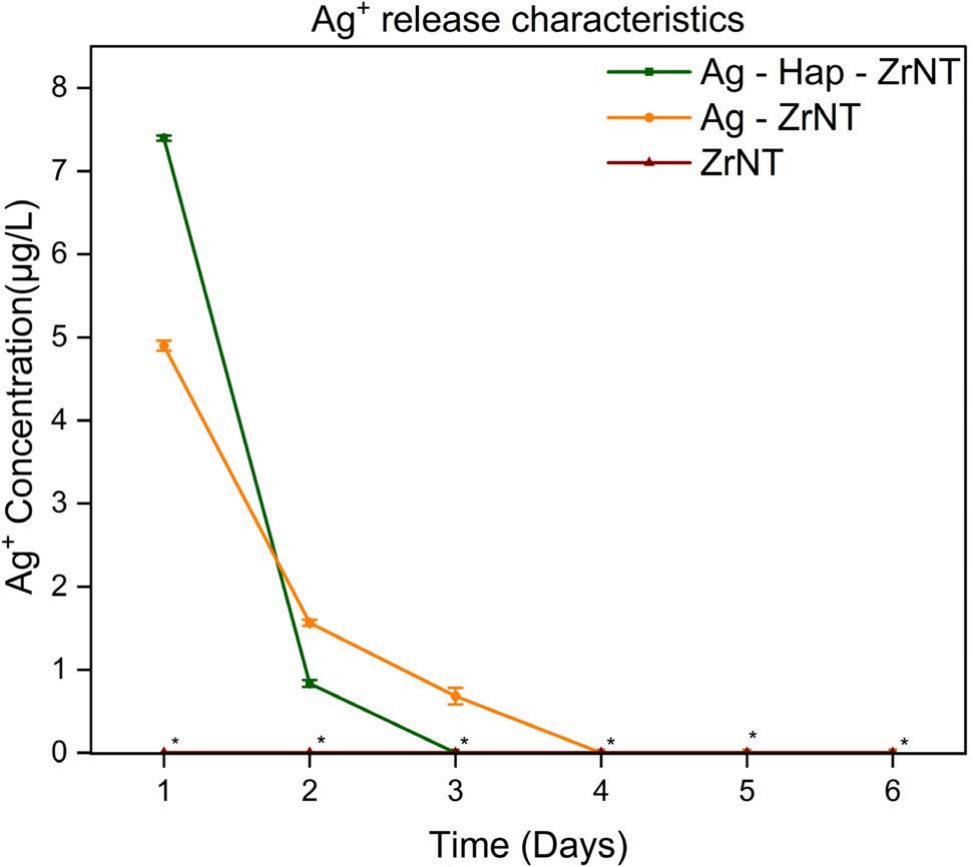
ICP-MS measurement monitoring the ion release behavior of Ag^+^ from Hap-Ag-ZrNT and Ag-ZrNT. *Values below the limit of quantification (LOQ).

The antibacterial activity of Ag-ZrNTs is primarily related to the release of Ag^+^ ions. To provide insight into the effect of Hap modification on the latter, Ag^+^ ion release was quantified using ICP-MS. As observed in Fig. 11, Ag^+^ was released from Hap-Ag-ZrNTs, particularly within the initial 24 h. Beyond this period, Ag^+^ release could not be reliably monitored because the concentration after the third day was below the limit of quantification. However, the total released amount over the 6 days testing period was comparable for both Ag-ZrNT and Hap-Ag-ZrNT, indicating that the Ag release is not suppressed by the Hap coating and, consequently, antibacterial properties of the Ag-ZrNT are expected to be maintained.

The reduction in concentration of released silver ions after the third day may also be partially responsible for the enhancement of cytocompatibility. However, a similar trend in the release profile was also observed for Ag-ZrNT, which showed enhanced cytotoxicity. While the Ag-release seems not to be altered drastically with the Hap-NP coating, CLSM images suggest that osteoblast adhesion and spreading preferentially occur in regions where Hap is present on the nanotube surface, indicating a locally enhanced cytocompatibility environment.

## 4 Conclusions

Conclusively, we successfully fabricated well-ordered zirconia nanotube (ZrNT) arrays and partially coated them with hydroxyapatite nanoparticles (Hap-ZrNT), preserving the nanotubular morphology. Surface analysis by XPS and ToF-SIMS/PCA confirmed the presence of hydroxyapatite and differentiated Hap-ZrNT from unmodified ZrNT, with characteristic calcium and phosphate signals.

Protein adsorption studies revealed that the surface morphology of ZrNT influences protein denaturation and orientation, while the detection of RGD-related fragments by Tof-SIMS offers a reliable marker for fibronectin adsorption. Notably, fibronectin adsorption, and thereby potential cell adhesion, was significantly enhanced by a Hap coating.

A biological assay, including confocal fluorescence imaging and XTT viability tests, confirmed that Hap-ZrNT significantly improve initial osteoblast adhesion and proliferation compared to unmodified ZrNT. Furthermore, coating Hap on Ag-ZrNT was found to mitigate silver-induced cytotoxicity.

The incorporation of Hap on the Ag-ZrNT does not significantly influence the silver ion release. Whether the previously reported antibacterial properties of the Ag-ZrNT can be fully retained, however, will be subject to future research.

Overall, this study introduces a versatile zirconia nanotube platform with improved early-stage cytocompatibility upon hydroxyapatite modification, while preserving the open nanotubular structure and still allowing release from the nanocontainers. This strategy maintains the potential for further functionalization, such as the loading of therapeutic agents into the ZrNT, which provides a foundation for future studies aimed at achieving multifunctionality for implant surfaces.

## Author contributions

GO – conceptualization, investigation, data curation, formal analysis, writing – original draft, LY – investigation, data curation, formal analysis, writing – review & editing, NF – investigation, data curation, formal analysis, writing – review & editing, SO – investigation, writing – review & editing, PCZF – investigation, CE – validation, review & editing, SJ – investigation, data curation, formal analysis, CP – investigation, formal analysis, writing – review & editing, HvdM – investigation, validation, supervision, writing – review & editing, MSK – funding acquisition, formal analysis, supervision, writing – review & editing.

## Conflicts of interest

The authors report no conflicts of interest.

## Supplementary Material

RA-016-D6RA00504G-s001

## Data Availability

Data for this article, including (SEM images, processed XPS and ToF-SIMS spectra, cell studies data), are available at Zenodo at (https://doi.org/10.5281/zenodo.17208984) and from the authors upon request. Supplementary information (SI): additional figures and datasets, including supplementary SEM images, ToF-SIMS spectra, PCA analyses, XRD and complementary cell study results that support the findings presented in the article. See DOI: https://doi.org/10.1039/d6ra00504g.
