# Supplementary material for: Synergistic surface modification of zirconia nanotubes with silver and hydroxyapatite for enhanced early-stage cytocompatibility: ToF-SIMS insights into fibronectin adsorption
Source: RSC Adv. 2026 Jul 2;16(34):32107–22. doi: 10.1039/d6ra00504g (PMC13325329; doi:10.1039/d6ra00504g)
Supplement: RA-016-D6RA00504G-s001 [file RA-016-D6RA00504G-s001.pdf]

Supplementary Information (SI) for RSC Advances.
This journal is © The Royal Society of Chemistry 2026

Supporting Information

Synergistic Surface Modification of Zirconia Nanotubes with Silver and Hydroxyapatite for Enhanced Early-Stage Cytocompatibility: ToF-SIMS Insights into Fibronectin Adsorption.

Gabriel Onyenso[a], Lu Yuan[b], Nastaran Farahbakhsh[a], Setareh Orangpour[a], Paola Carolina Zamudio Franco,[a] Johannes Schmitt,[c, d] Carsten Engelhard,[c, d,e] Christian Pritzel[a], Henny C. van der Mei[b], Manuela S. Killian[a,e]

(a) Chemistry and Structure of Novel Materials, Department of Chemistry & Biology, University of Siegen, Paul-Bonatz-Str. 9-11, 57076 Siegen, Germany

(b) University of Groningen and University Medical Center of Groningen, Department of Biomaterials & Biomedical Technology, Antonius Deusinglaan 1, 9713 AV Groningen, the Netherlands

(c) Federal Institute for Materials Research and Testing (BAM), Richard-Willstätter Str. 11, D-12489 Berlin, Germany

(d) Analytical Chemistry, Department of Chemistry & Biology, University of Siegen, Adolf-Reichwein-Str. 2, 57076 Siegen, Germany.

(e) Center of Micro- and Nanochemistry and (Bio-)Technology (Cµ), Department of Chemistry-Biology, University of Siegen

Characterization of zirconia compact oxide and nanotubes modified with silver and hydroxyapatite nanoparticles

The zirconia compact oxide displays a flat oxide layer with no nanotubular structure observed, **Figure SI 1**.

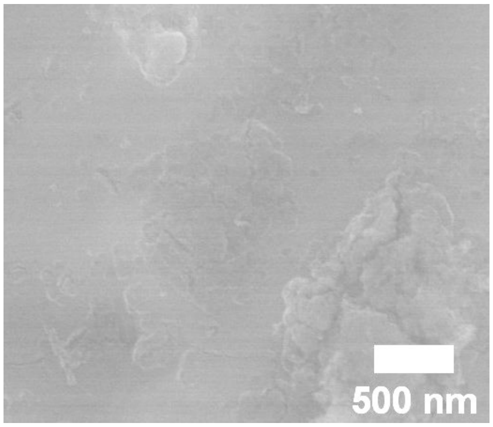

Figure SI 1: SEM top-view image of zirconia compact oxide (ZrCo).

Figures SI 2 and S 3 show selected regions of the ToF-SIMS spectra of ZrNT and Hap-ZrNT. Both fragments characteristic of the ZrNTs (ZrO^+ and ZrO^-), which are dominant on the unmodified ZrNTs, and Hap (Ca^+, CaO^+, $CaOH^+$, PO^-, PO_2^-, PO_3^-), which are only found on the Hap modified sample, are depicted. This demonstrates that

neither contaminations of the ZrNTs with Ca- or P-based compounds, nor conflicting fragments in these regions of interest are present.

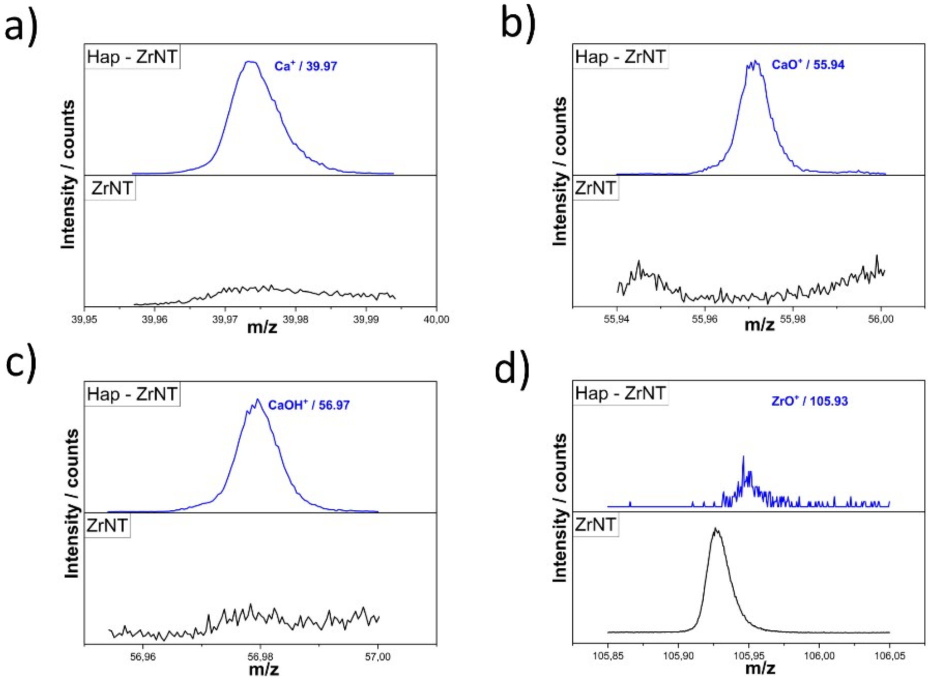

*Figure SI 2: Tof-SIMS spectra of specific hydroxyapatite-related positive fragments of hydroxyapatite-modified zirconia nanotubes (Hap–ZrNT) and bare zirconia nanotubes (ZrNT). a) m/z 39.97 (Ca^+)**,** b) m/z 55.94 (CaO^+), c) m/z 56.97 ($CaOH^+$), d) Tof-SIMS spectra of substrate signal m/z 105.93 (ZrO^+)**.***

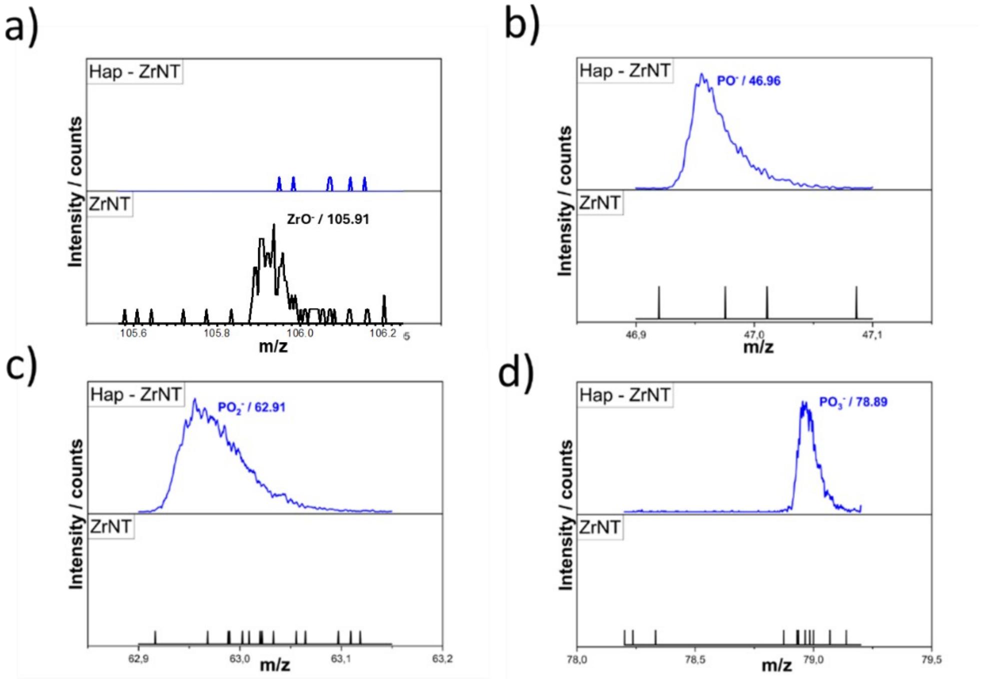

Figure SI 3: ToF – SIMS spectra of specific hydroxyapatite-related negative fragments of hydroxyapatite-modified zirconia nanotubes (Hap–ZrNT) and bare zirconia nanotubes (ZrNT). a) m/z 105.91 ($ZrO^−$), b) m/z 46.96 ($PO^−$), c) m/z 62.97 ($PO_2^−$), d) m/z 78.89 ($PO_3^−$).

BSA adsorption on zirconia nanotubes at a lower concentration

Tof-SIMS PCA was used to evaluate the BSA adsorption on zirconia substrate (ZrNT and ZrCo) at a lower concentration (50 µg/mL). This analysis aimed to determine whether the protein concentration influences adsorption and whether ToF-SIMS/PCA can differentiate the adsorption characteristics based on concentration differences. **Figure SI 4** confirms that, even at lower concentration, ToF-SIMS/PCA can distinguish between the substrates, consistent with the results observed at higher concentration.

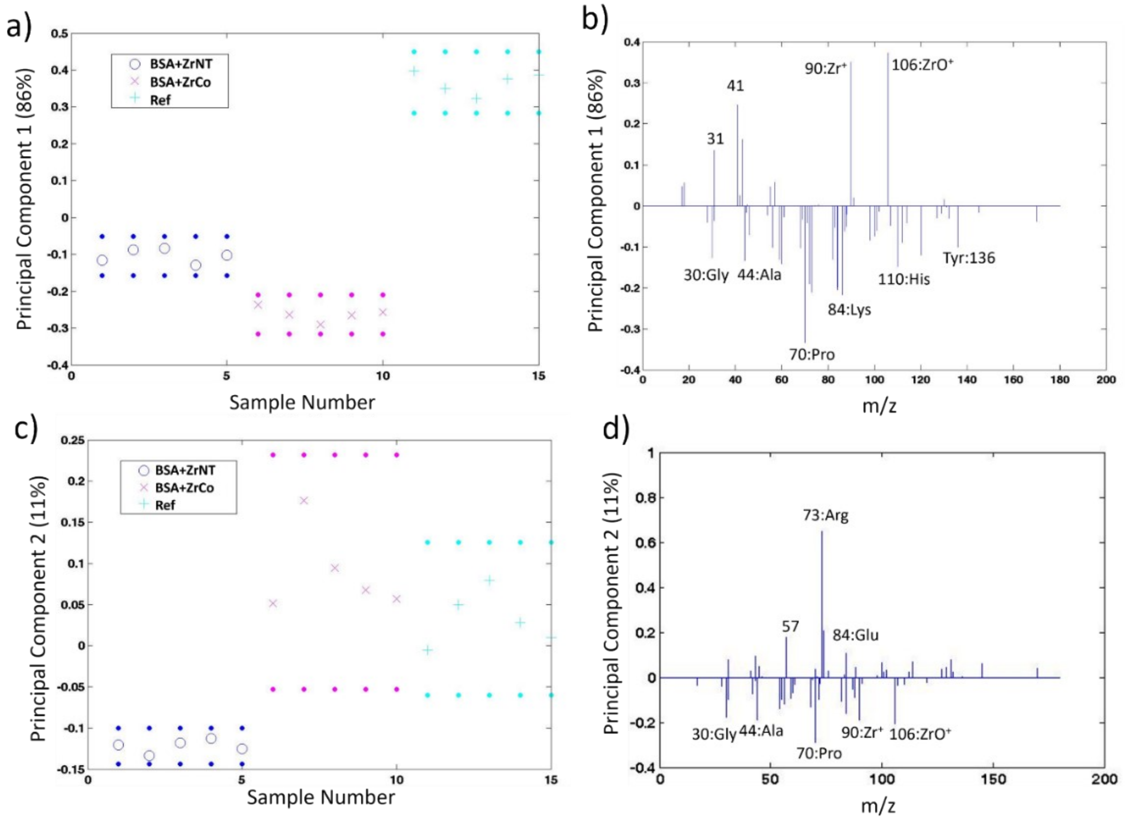

Figure SI 4: a) and c) Scores plot from PCA 1 and 2 of the positive ion spectra of bovine serum albumin (BSA) adsorption on zirconia nanotube (ZrNT) and zirconia compact oxide (ZrCo) from 50 µg/mL protein solution. b) and d) The corresponding loading plots. The dotted lines in the scores plot show the 95% confidence limits. Ref stands for the reference sample (bare substrate without protein).

Fibronectin adsorption and identification using ToF-SIMS and PCA

50 µg/mL of fibronectin bovine plasma (FBP) was adsorbed on zirconia nanotubes (ZrNT) and zirconia compact oxide (ZrCo). **Figure SI 5** shows the PCA of the positive ion Tof-SIMS spectra obtained from the adsorbed protein films. The scores plot for PC 1 & 2 captures 96 % of the total variance in the dataset. PC 1 distinguishes between the protein-adsorbed substrate and the reference sample (zirconia without protein). The corresponding loading plot shows amino acid fragment ion features predominantly on the protein-modified substrate, while the substrate signals (Zr^+ and ZrO^+) contribute mainly to the reference sample. Additionally, fragments not directly related to individual amino acids but from the Arginyl-glycyl-aspartic acid (RGD) peptide motif, common in cell adhesion proteins such as fibronectin, were also observed on the protein-adsorbed substrate. This is noteworthy, as these RGD-related fragments can serve as ToF-SIMS unique molecular markers for identifying adhesive proteins and distinguishing them from non-adhesive counterparts that do not contain this peptide motif.

PC 2 primarily differentiates between the spectra of ZrNT and ZrCo. The loadings plot indicates that amino acid peaks such as $CH_3N_2^+$ (43.03, Arg), $C_2H_7N_3^+$ (73.06, Arg), and the RGD-related signal ($C_{13}H_{12}N_3O_2^+$ / 122.09) feature more on the ZrCo. It should be noted $C_{13}H_{12}N_3O_2^+$ / 122.09 signal originates mainly from the Arginine residue of the RGD motif, and if the arginine fragment is more prominent on the compact oxide, it may impact the RGD–122 signal. However, the other RGD-related peaks, along with amino acids such as $C_4H_8N^+$ (70.06, Pro), $C_2H_6N^+$ (44.05, Ala), $C_5H_{10}N^+$ (84.08, Lys), and $C_9H_8N^+$ (130.07, Trp), contribute significantly to the ZrNT spectra.

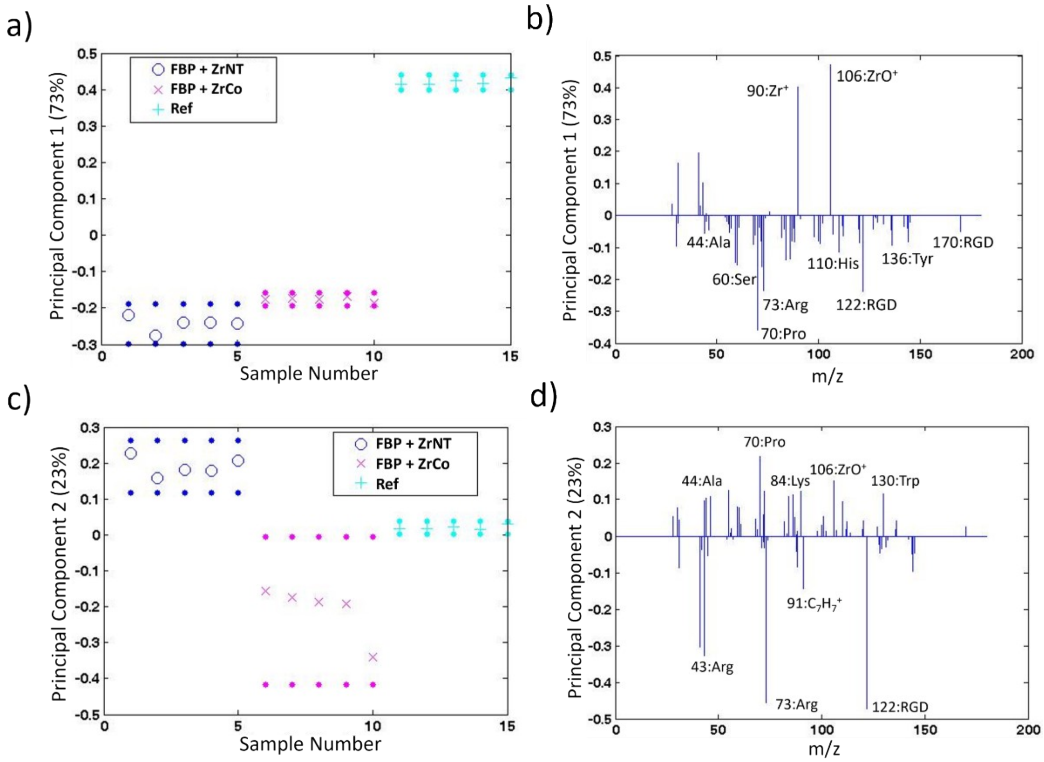

Figure SI 5: a) and c) Scores plot from PCA 1 and 2 of the positive ion spectra of fibronectin bovine plasma (FBP) adsorption on zirconia nanotubes (ZrNT) and zirconia compact oxide (ZrCo) from 50 µg/mL protein solution; b) and d) corresponding loading plots. The dotted lines in the scores plot show the 95% confidence limits. Ref stands for the reference sample (bare substrate without protein).

XRD characterization of hydroxyapatite-modified zirconia nanotubes

The fabricated samples were characterized using X-ray diffraction (XRD) to determine their crystalline structure. **Figure SI 6** compares the XRD patterns of hydroxyapatite nanoparticles (Hap NP), zirconia nanotube modified with hydroxyapatite nanoparticles (Hap–ZrNT), and the unmodified zirconia nanotubes (ZrNT). The ZrNT sample displays diffraction peaks mainly from the monoclinic zirconia crystalline phase at 24˚, 28˚, 31˚, and 50˚[73]. In contrast, the Hap–ZrNT sample exhibits additional peaks at 2θ = 26˚, 31˚, and 33˚, which fit well with the pattern observed on the Hap NP[74]. These peaks are characteristic of monoclinic hydroxyapatite and are absent in the unmodified ZrNT, confirming the successful surface modification with hydroxyapatite nanoparticles. These results further support the findings from the XPS and Tof-SIMS analyses.

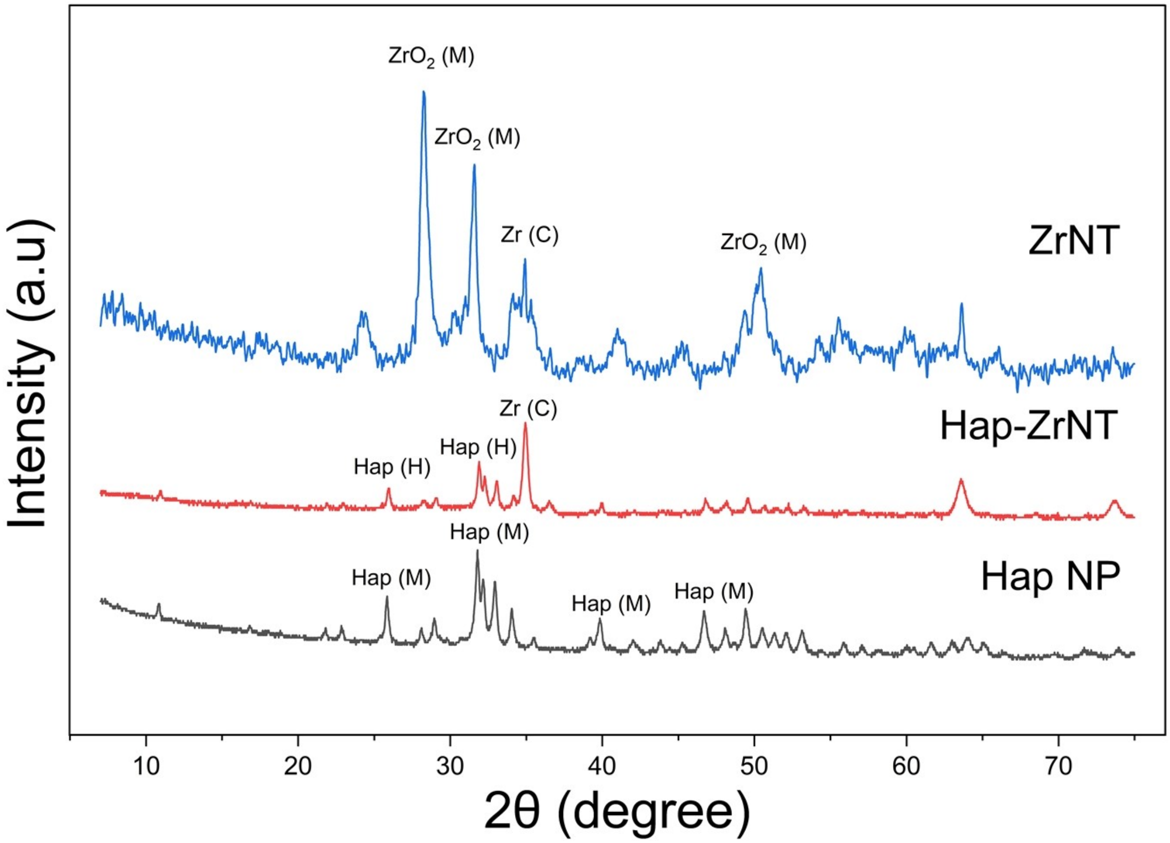

Figure SI 6: XRD spectra of hydroxyapatite nanoparticles (Hap NP), zirconia nanotubes modified with hydroxyapatite nanoparticles (Hap-ZrNT), and unmodified zirconia nanotubes (ZrNT).

Cell studies on zirconium and zirconium-silver alloy metal foils

The zirconium foil and zirconium–silver alloy were investigated to determine their cytotoxicity and viability of the non-anodized materials. As observed in the fluorescence confocal images below, both materials demonstrated good biocompatibility, exhibiting good cell adhesion after 24 h and proliferation by day 3. Furthermore, they were chemically and structurally stable under the culture conditions.

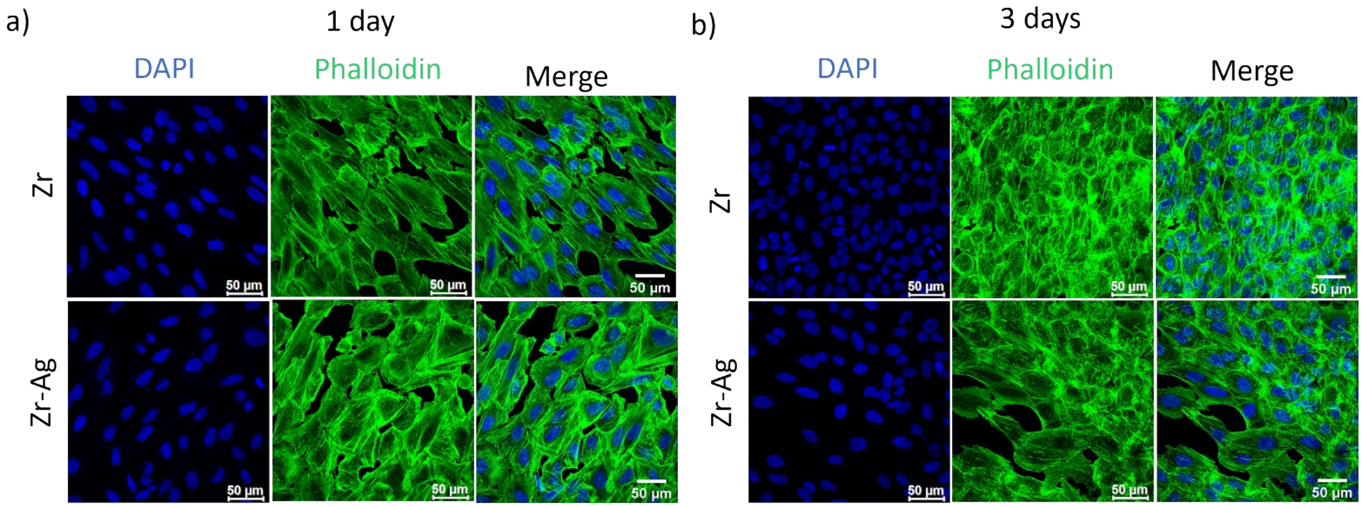

Figure SI 7: Fluorescence confocal microscope images (blue, nucleus; green, F-actin) of osteoblast cells on non-anodized zirconium foil (Zr) and zirconium–silver alloy (Zr-Ag) surfaces after cell seeding for 24 h a) and 3 days b).

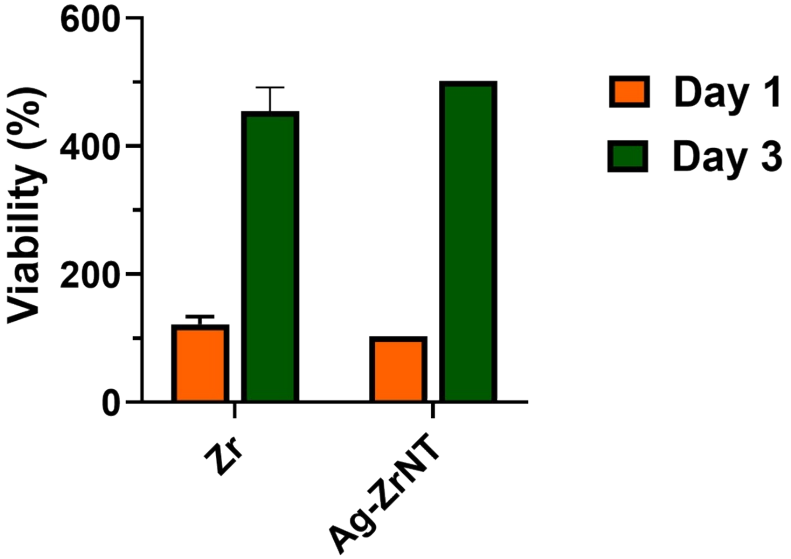

Figure SI 8: Cell viability of osteoblasts cultured on Zr foil and Ag-Zr alloy foil (not anodized) for 1 and 3 days (s). Data represent the mean ± s.d. (n=3).
